# *In vivo* monitoring of polymorphonuclear myeloid-derived suppressor cells in experimental rheumatoid arthritis

**DOI:** 10.3389/fimmu.2026.1909885

**Published:** 2026-10-15

**Authors:** Johannes Schwenck, Kerstin Fuchs, Mohamed Ali Jarboui, Sophia Geiger, Marc Neumann, Hasan H. Öz, Melanie Carevic, Sabrina Hoffmann, Dominik Hartl, Christoph M. Griessinger, Irene Gonzalez-Menendez, Leticia Quintanilla-Martinez, Jörg Henes, Gerald Reischl, Andreas Maurer, Bernd J. Pichler, Manfred Kneilling

**Affiliations:** 1 Werner Siemens Imaging Center, Department of Preclinical Imaging and Radiopharmacy, Eberhard Karls University, Tuebingen, Germany; 2 Department of Nuclear Medicine and Clinical Molecular Imaging, Eberhard Karls University, Tuebingen, Germany; 3 Cluster of Excellence iFIT (EXC 2180) “Image-Guided and Functionally Instructed Tumor Therapies”, Eberhard Karls University, Tuebingen, Germany; 4 Medical Proteome Center, Division for Experimental Ophthalmology, Institute for Ophthalmic Research, Eberhard Karls University, Tuebingen, Germany; 5 Department of Pediatrics, Eberhard Karls University, Tuebingen, Germany; 6 Department of Dermatology, Eberhard Karls University, Tuebingen, Germany; 7 Institute of Pathology and Neuropathology, Eberhard Karls University, Tuebingen, Germany; 8 Core Facility Histology, Faculty of Medicine, University Hospital Tübingen, Eberhard Karls University Tübingen, Tübingen, Germany; 9 Department of Internal Medicine 2, University of Tubingen, Tubingen, Germany

**Keywords:** arthritis (including rheumatoid arthritis), cell tracking and monitoring, MDSC (myeloid-derived suppressor cells), PET/MRI, proteomics, molecular imaging, myeloid cells

## Abstract

**Introduction:**

Myeloid-derived suppressor cells (MDSCs) regulate immune responses, but their role in autoimmune diseases such as rheumatoid arthritis (RA) remains incompletely understood. Here, we applied non-invasive positron emission tomography (PET) imaging to investigate the migration dynamics of polymorphonuclear (PMN)-MDSCs in experimental RA.

**Materials and methods:**

RA was induced in female BALB/c mice via K/BxN serum. Immune infiltrates in arthritic ankles were characterized by flow cytometry and liquid chromatography–mass spectrometry (LC–MS)-based proteomics. We radiolabeled bone marrow-derived (BM-PMN-MDSCs) and RA ankle-derived PMN-MDSCs (RA-PMN-MDSCs) intracellularly with [^64^Cu]Cu-PTSM or Ly6G-specific [^64^Cu]Cu-NOTA-1A8. Radiolabeled PMN-MDSCs were adoptively transferred into RA mice and tracked longitudinally by PET/MRI. In a second cohort, [^64^Cu]Cu-NOTA-1A8 was injected directly for *in vivo* imaging of Ly6G^+^ myeloid cells over 48 h.

**Results:**

RA progression was associated with a marked increase in the number of CD11b^+^Ly6C^med^Ly6G^+^ PMN-MDSCs in arthritic ankles, whereas the number of CD11b^+^Ly6C^high^Ly6G^−^ monocytic-MDSCs remained unchanged. PET/MRI demonstrated significantly enhanced homing of RA-PMN-MDSCs to inflamed ankles compared with that of BM-PMN-MDSCs, indicating disease-associated functional adaptation. Direct *in vivo* targeting of MDSCs with [^64^Cu]Cu-1A8 revealed strongly increased uptake in arthritic ankles at 1 h, 24 h, and 48 h *p.i.*, which was significantly reduced by Ly6G blockade, confirming tracer specificity. Proteomic analysis revealed enrichment of extracellular matrix organization and integrin signaling pathways in RA-PMN-MDSCs, suggesting that a distinct inflammatory signature underlies their migratory behavior.

**Conclusion:**

PET/MRI enables non-invasive monitoring of PMN-MDSC migration during experimental RA and provides novel insights into the role of these cells in autoimmune inflammation, supporting the development of PMN-MDSCs as biomarkers and therapeutic targets in autoimmune diseases.

## Introduction

Myeloid-derived suppressor cells (MDSCs) are a heterogeneous population of immature myeloid cells characterized by their effects on tumor immunity via T-cell suppression (1–3). Although the functional importance of MDSCs as immunosuppressive cells in the tumor microenvironment is quite evident and their regulatory influences on immune responses such as sepsis (4) or graft-versus-host reactions (5) have been described, the distinct roles of MDSCs and their subsets in the dynamic mechanisms of autoimmune diseases are still not understood in depth (6–9).

Two MDSC subsets have been reported to display different characteristics in mice and humans. Polymorphonuclear (PMN)-MDSCs in mice were defined by flow cytometry as CD11b^+^Ly6G^+^Ly6C^low^, whereas mononuclear MDSCs (M-MDSCs) were defined as CD11b^+^Ly6G^-^Ly6C^high^ (1). In human samples, these cells are most commonly defined as CD14^-^ CD11b^+^CD33^+^Lin^-^HLA-DR^-^ cells (M-MDSCs) and CD11b^+^CD14^−^CD15^+^/CD66b^+^ low-density cells (PMN-MDSCs) (1). Although distinguishing PMN-MDSCs from neutrophils can be difficult, the function of PMN-MDSCs clearly differs from that of neutrophils because they are able to suppress T-cell activation (1).

Rheumatoid arthritis (RA) is among the most common systemic autoimmune diseases and has an unknown etiology and limited therapeutic options (10). Local inflammation in arthritic joints attracts immune cell populations in an antigen-unspecific manner. This process is essentially driven by a chemotactic gradient and by the increased expression of endothelial adhesion molecules within or near the site of inflammation. Therefore, the selective recruitment of distinct immune cell populations is a prerequisite for the onset of inflammatory diseases such as RA and is essential for inducing local inflammation in the affected tissue (11).

Zhang et al. found that the number of PMN-MDSCs significantly increased in an experimental model of collagen-induced arthritis, promoting Th17 cell differentiation and playing a proinflammatory role during disease progression (12). Furthermore, M-MDSCs are more prevalent at late stages of the disease (12). As depletion of PMN-MDSCs in the early stages of arthritic joint inflammation ameliorates joint destruction (12), depletion or inhibition of PMN-MDSC functions might represent a novel and innovative treatment approach for RA. This is particularly important because PMN-MDSCs are considered a major source of interleukin-1β (8). Nevertheless, findings from other groups also suggest that MDSCs play anti-inflammatory roles, including the induction of regulatory T cells (Tregs) and the inhibition of Th17 cells (8). Therefore, innovative strategies are needed to elucidate the controversial role of MDSCs *in vivo*.

The K/BxN serum transfer model of RA is a well-established, T- and B-cell-independent murine model that allows us to study the immunological mechanisms of the RA effector phase (13, 14). Thus, the inflammatory response is driven by autoantibodies (Abs) against glucose-6-phosphate isomerase (GPI), which leads to the formation of immune complexes, complement C5a formation, macrophage and mast cell activation, the homing of neutrophils and, consequently, arthritic joint inflammation (15). As this model mimics the effector phases of RA independent of the adaptive immune response, it represents an ideal tool for studying potential new therapeutic targets in the preclinical setting (15). While many research groups have focused on investigating the role of PMN-MDSCs in the development of collagen-induced arthritis (16–18), the specific role of MDSCs in the effector phase of K/BxN-induced arthritis has not been thoroughly investigated.

Non-invasive imaging is an emerging toolbox for tracking cell migration kinetics and homing patterns *in vivo* (19). Griessinger et al. established an efficient and reliable method to radiolabel murine lymphocytes and track their homing patterns *in vivo* using positron emission tomography (PET) (19). More recently, we established *in vivo* tracking of MDSCs by PET in an experimental tumor model (20).

The aim of our study was to elucidate the homing dynamics of PMN-MDSCs and BM-MDSCs in inflamed arthritic ankles non-invasively *in vivo* and to develop effective *in vivo* tracking methods for MDSCs, which are needed for monitoring MDSCs as potential biomarkers and treatment targets in preclinical research and clinical translation. More specifically, we aimed to identify phenotypic differences in the immigration into inflamed arthritic ankles between inflamed ankle-derived PMN-MDSCs and “naive” bone marrow-derived PMN-MDSCs.

## Materials and methods

### Animals

Female 8–12-week-old BALB/c mice were purchased from Charles River Laboratory (Sulzfeld, Germany) and kept under specific pathogen-free conditions in individually ventilated cages. All animal experiments were approved by the Regierungspräsidium Tübingen, Germany (R 3/15 and R 3/23).

### Induction of RA

K/BxN serum with Abs against GPI was derived from K/BxN transgenic mice, diluted 1:1 with PBS and injected intraperitoneally (200 µL *i.p.*) into BALB/c mice on Day 0. The timeline of RA induction and PET investigations is shown in **Supplementary Figure 1**. The mice that were used for the isolation of MDSCs from inflamed ankles were injected with K/BxN serum on Days 0 and 2. Control mice were injected with an equal volume of serum derived from healthy C57BL/6 mice. Ankle swelling was monitored using a caliper (Oditest micrometer, Kroeplin, Schlüchtern, Germany) to assess the severity of arthritis (13).

### Bone marrow-derived and RA ankle-derived MDSCs

BM-derived MDSCs were isolated from the femur and the tibia according to Marigo et al. (21) and cultured overnight on UV-irradiated bacterial culture dishes in 5 mL of Roswell Park Memorial Institute 1640 (RPMI) medium. The cells were transferred on Day 1 to cell culture plates (Sarstedt, Nümbrecht, Germany) in 10 mL of RPMI medium supplemented with 40 ng/mL granulocyte macrophage-colony stimulating factor (GM-CSF) and IL-6 (Peprotech, Hamburg, Germany). Cytokines were added on Days 3 and 5 of the cell culture. CD11b^+^ Ly6G^+^ Ly6C^+^ PMN-MDSCs were isolated by magnetic cell sorting (MACS; Miltenyi Biotec, Bergisch Gladbach, Germany).

The cells from the RA ankle lavage were derived using 1 mL of phosphate-buffered saline (PBS) and 10% fetal calf serum (FCS) on Day 6 after the induction of experimental arthritis. Ankle lavage was performed as a terminal procedure. The number of PMN-MDSCs derived from inflamed arthritic ankles of experimental mice was relatively low. We were able to obtain approximately 5 × 10^5^ PMN-MDSCs from one inflamed ankle. Therefore, we pooled PMN-MDSCs from 5–8 experimental RA mice for each *in vitro* RA-MDSC study and RA-MDSC labeling experiment. Finally, CD11b^+^ Ly6G^+^ Ly6C^+^ PMN-MDSCs were isolated by magnetic cell sorting (MACS; Miltenyi Biotec, Bergisch Gladbach, Germany).

### NOTA conjugation and radiolabeling of the 1A8 mAb

The 1A8 mAb (6.3 mg) was dissolved in 0.1 M HEPES buffer (pH 7.5) containing 0.5 M NaCl, and 1.5 mg of NOTA-NHS (Chematech, Dijon, France; 55-fold molar excess over the antibody) was added (freshly dissolved in water, 10 mg/mL). All buffers were treated with Chelex 100 (sodium form; Sigma–Aldrich) to minimize metal contamination.

After incubation overnight at 4 °C, the unreacted chelator was removed by multiple rounds of ultrafiltration (Amicon Ultra-15, 30 kDa; Merck Millipore) using Chelex-treated 0.25 M sodium acetate (pH 6) and Amicon Ultra-15 tubes (30 kDa).

The radioisotope ^64^Cu was produced as previously described (19) and was buffered to pH 5–6 with 0.5 M ammonium acetate for antibody radiolabeling. The antibody was added (600 µg per 300 MBq) and incubated for 60 min at 42 °C. The labeling reaction was quenched with DTPA, and the radiochemical purity was assessed by TLC on silica plates (Polygram SIL G/UV254, Macherey-Nagel; 0.1 M sodium citrate, pH 5).

### Analysis of PMN-MDSC viability

To examine the impact of [^64^Cu]Cu-PTSM and [^64^Cu]Cu-1A8-mAb-labeling on PMN-MDSC viability, the viability of [^64^Cu]Cu-PTSM-labeled, [^64^Cu]Cu-1A8-mAb-labeled, or unlabeled PMN-MDSCs derived from arthritic ankles or from the BM of experimental mice was assessed by flow cytometry analysis 24 and 48 h after the labeling procedure.

Given that the number of PMN-MDSCs derived from inflamed arthritic ankles of experimental mice was relatively low (approximately 5 × 10^5^ PMN-MDSCs from one inflamed ankle), PMN-MDSCs from 5–8 experimental RA mice were pooled to comprehensively analyze 2 × 10^6^ PMN-MDSCs per experimental condition. To assess the impact of [^64^Cu]Cu-PTSM and [^64^Cu]Cu-1A8-mAb labeling, 2 × 10^6^ MDSCs were dispersed on a 24-well plate in 1 mL per well and investigated 24 and 48 h later. PMN-MDSCs were stained with 7-aminoactinomycin D (7-AAD) and PE-annexin V using the PE Annexin V Apoptosis Detection Kit I (BD Biosciences) according to the manufacturer’s instructions.

### *In vivo* depletion of MDSCs

For MDSC depletion, 100 µg of RB6-8C5 or LTF2 isotype control antibody was injected *i.p.* 24 h before PET/MRI on Day 5 after arthritis induction.

### Carboxyfluorescein succinimidyl ester suppression assay

To prove the functionality of the PMN-MDSCs isolated from RA ankles, a CFSE dilution assay was performed as described previously (22). In brief, CD11b^+^Ly6G^+^ PMN-MDSCs were isolated from the lavage of RA ankles by using MACS (MDSC isolation kit; Miltenyi Biotec, Germany) and were cocultured for 3 days (37 °C, 5% CO_2_) with MACS-sorted CFSE-stained CD4^+^ T cells from the spleen. T cell:MDSC ratios of 1:1, 1:2, 1:4, and 1:8 were investigated. The number of T cells per well was 10^5^, and MDSCs were added accordingly. T-cell proliferation was induced by CD3/CD28 beads (mouse T-cell activation kit; Miltenyi Biotec, Germany) and recombinant mouse IL-2 (50 U/ml; Biolegend). CFSE fluorescence intensity was analyzed by flow cytometry to determine the percentage of polyclonally proliferating T cells. For graphical visualization, the data were normalized to the proliferation of the stimulated control T cells without the addition of MDSCs.

### Flow cytometry

Flow cytometry analyses were performed on a BD LSRII flow cytometer (BD Biosciences, Franklin Lakes, New Jersey, United States), and the data were analyzed with FlowJo (FlowJo LLC, Ashland, Oregon, USA) as described previously (13). The antibody panel used for flow cytometry is summarized in **Supplementary Table 1**. If the number of cells isolated from the ankles was very low, the cells from both hind legs were pooled. For analysis of PMN-MDSC viability, PMN-MDSCs were stained with 7-aminoactinomycin D (7-AAD) and PE-annexin V using the PE Annexin V Apoptosis Detection Kit I (BD Biosciences) according to the manufacturer’s instructions. The FACS gating strategy is shown in **Supplementary Figure 3**.

### Cell labeling using [^64^Cu]Cu-NOTA-1A8 and [^64^Cu]Cu-PTSM

For radiolabeling, 20 µL of 0.7 MBq [^64^Cu]Cu-NOTA 1A8 or [^64^Cu]Cu-PTSM was added to 10^6^ PMN-MDSCs and incubated for 30 min at 37 °C as described by Hoffmann et al. and Griessinger et al. (19, 20, 23). The cells were subsequently washed and suspended in PBS for adoptive transfer into experimental mice. Approximately 50% of the PMN-MDSCs were lost during the labeling procedure. After washing and resuspending of the cells in PBS, the viable cells were counted, and 0.5 µCi (0.185 MBq) of the radiolabeled PMN-MDSCs was injected intravenously (*i.v.)* (corresponding to 2–3 × 10^6^ radiolabeled PMN-MDSCs) into the experimental mice.

### [^64^Cu]Cu-NOTA-1A8 and [^64^Cu]Cu-PTSM cell radiolabeling stability

We transferred 10^5^ PMN-MDSCs in 1 mL of medium into dedicated gamma-counter tubes directly after radiolabeling. The labeling stability of [^64^Cu]Cu-NOTA-1A8 and [^64^Cu]Cu-PTSM was determined 5 h, 24 h, and 48 h after the initial radiolabeling using a gamma counter (PerkinElmer).

The ^64^Cu activity was measured in the cell pellet and in the supernatant using a gamma counter. The amount of free Cu was not determined. The labeling stability was referred to the stability of the cellular radiolabeling of the PMN-MDSCs but not to the radiolabeled antibody.

### *In vivo* PET imaging and analysis

*In vivo* PET was performed using a dedicated small animal PET scanner (Siemens Medical Solutions, Inc., Knoxville, TN, USA) as described previously (13). [^64^Cu]Cu-PTSM-PMN-MDSCs (n = 3) and [^64^Cu]Cu-NOTA-1A8-PMN-MDSCs (n = 3) were injected *i.v.*, and static PET scans were acquired 1 h and 24 h after injection. The [^64^Cu]Cu-NOTA-1A8 tracer was injected *i.v.*, and static PET scans were acquired 1 h, 24 h, and 48 h after injection (n = 3), as indicated in **Supplementary Figure 2**. We applied approximately 10 MBq of [^64^Cu]Cu-1A8 mAbs, corresponding to a dose of 20 µg of the mAb.

Additionally, sequential MRI scans were acquired for anatomic correlation, except at 48 h *p.i.* for the [^64^Cu]Cu-NOTA-1A8 tracer, as the MRI machine was not available on this day. To analyze the uptake in the ankles, the average uptake in both hindleg ankles was calculated. We analyzed the data by drawing a defined region of interest of 3.5 mm in diameter on three adjacent image slices of the normalized PET images. The regions of interest covered the area of maximum activity in the inflamed and healthy ankles.

### Liquid chromatography–mass spectrometry

MDSCs collected from the ankle lavage of GPI-induced RA mice or bone marrow of control mice (5 biological replicates for each experimental group) were lysed for 10 min at 4 °C in 1% NP40, 20 mM Tris-HCl (pH 7.5), 150 mM NaCl, and 1 mM MgCl2 supplemented with protease inhibitor tablets (Roche, Basel, Switzerland) and phosphatase inhibitors (2 mM sodium orthovanadate, 10 mM sodium fluoride, and 10 mM β-glycerophosphate; all from Sigma–Aldrich, St. Louis, USA). The lysates were cleared of debris by centrifugation at 20,000 g at 4 °C for 10 min. The total protein concentration in the supernatants was measured by the Pierce BCA assay according to the manufacturer’s instructions. Equal amounts of total protein from each biological replicate were digested with trypsin in 2 M urea, 50 mM Tris-HCl (pH 7.5), and 1 mM DTT supplemented with 5 μg/mL modified sequencing-grade trypsin (Promega, Fitchburg, USA) overnight at 28 °C. Peptides were alkylated using iodoacetamide (5 mg/mL) and incubated in the dark for 30 min at room temperature. The samples were desalted using C18 Stage Tips as previously described (24) and analyzed by liquid chromatography–mass spectrometry.

The protein abundance was analyzed on an Ultimate3000 RSLC system connected to a Q-Exactive plus Orbitrap mass spectrometer (Thermo Fisher Scientific, Waltham, USA). Tryptic peptides were loaded onto a nanotrap column (300 μm × 5 mm precolumn, packed with Acclaim PepMap100 C18, 5 μm, 100 Å; Thermo Scientific) at a flow rate of 30 µL/min in 0.1% trifluoroacetic acid in HPLC-grade water. After 3 min, the peptides were eluted and separated on an analytical column (75 μm × 25 cm, Acclaim PepMap RSLC C18, 2 μm, 100 Å; Thermo Fisher Scientific) by a linear gradient from 2% to 30% buffer B (80% acetonitrile and 0.08% formic acid in HPLC-grade water) in buffer A (2% acetonitrile and 0.1% formic acid in HPLC-grade water) at a flow rate of 300 nL/min over 150 min. The remaining peptides were eluted by a short gradient from 30% to 95% buffer B for 10 min. The MS parameters were as follows: for full MS spectra, the scan range was 335–1,500 with a resolution of 120,000 at m/z=200. MS/MS acquisition was performed in top-speed mode with a 3-s cycle time. The maximum injection time was 50 ms. The AGC target was set to 400,000, and the isolation window was 1 m/z. Positive ions with charge states of 2–7 were sequentially fragmented by higher-energy collisional dissociation. The dynamic exclusion duration was set to 60 s, and the lock mass option was activated and set to a background signal with a mass of 445.12002.

### LC–MS data analysis and integration

PMN-MDSCs collected from the ankle lavage or BM of RA mice or control mice (5 biological replicates for each experimental group) were analyzed by LC–MS. Raw data collected from LC–MS runs were analyzed using MaxQuant Version 1.6.17.0 (25). The default search parameters were used. Proteins were identified with a minimum of 1 unique peptide per protein with an FDR < 0.01 at the protein and peptide level. The label-free quantification (LFQ) option was activated, and LFQ intensities were used to generate the quantitative proteomics data. Missing data imputation to improve proteome coverage and the reliability of peptide and protein quantification was carried out using IceR (26). For data integration and statistical analysis, Perseus software was used (27). Proteins were only quantified if they had a minimum of 1 unique peptide and 2 LFQ intensities out of 5 biological replicates in at least one experimental group (GPI-induced or control group). For differential protein abundance, an unpaired two-sided permutation-based FDR t-test was used (FDR < 0.05, 250 randomizations), and pathway analysis was carried out against the curated Reactome pathway using Proteomill (28). Immune cell profiling and distribution analysis were carried out using the Bigomics platform (https://github.com/bigomics/omicsplayground) against the LM22 signature matrix of 547 genes that distinguish among 22 mature human hematopoietic populations (29).

### *Ex vivo* biodistribution analysis

To validate the *in vivo* PET data, the biodistributions of [^64^Cu]Cu-PTSM- or [^64^Cu]Cu-NOTA-1A8 mAb-labeled RA-PMN-MDSCs or [^64^Cu]Cu-1A8-mAbs in organs/tissues of interest, namely, blood, lungs, liver, kidneys, spleen, muscle, paws, and ankles, were analyzed by γ-counting *ex vivo* after 24 h ([^64^Cu]Cu-PTSM-labeled RA- and BM-PMN-MDSCs; [^64^Cu]Cu-1A8-mAbs-labeled RA- and BM-PMN-MDSCs) or 48 h ([^64^Cu]Cu-1A8-mAbs) as previously described (20). For [^64^Cu]Cu-PTSM-labeled RA- and BM-PMN-MDSCs, lung tissue was not investigated. Briefly, retro-bulbar blood samples and organs of interest were collected from experimental mice directly after sacrifice by CO_2_ asphyxiation. A standard solution with a defined activity served as a reference for calculating the percentage of injected dose per gram of tissue (%ID/g). The tubes with the organs of interest were measured in a Wizard2 automated γ-counter (PerkinElmer) at an energy window of 350 to 650 keV. The same ankles that were analyzed by PET (ROIs; %ID/CC) were isolated and measured in the γ-counter. Therefore, the *in vivo* PET data (%ID/CC) should approximately match the *ex vivo* γ-counter measurements (%ID/g).

### Histological analyses

For histological analyses, 3–5 µm-thick sections were cut and stained with hematoxylin and eosin (H&E). Immunohistochemistry was performed on an automated immunostainer (Ventana Medical Systems, Inc.) according to the company’s protocols for open procedures with slight modifications. All the slides were stained with the antibody MPO (Abcam). Appropriate positive and negative controls were used to confirm the adequacy of the staining. The histologic samples were analyzed by an experienced pathologist (L. Quintanilla-Martinez). All the samples were scanned with a Ventana DP200 (Roche, Basel, Switzerland) and processed with the Image Viewer MFC application. Final image preparation was performed with Adobe Photoshop 2025.

### Statistical analysis

Quantitative data are reported as the mean ± standard deviation (SD) unless stated otherwise. For statistical comparison, we used a two-sample t-test calculated with the software JMP (SAS Institute Inc., Cary, NC, USA, version 9). P values <0.05 were considered to indicate statistical significance.

## Results

### Characterization of MDSCs in K/BxN serum-induced RA

Early onset and progression of K/BxN serum-induced RA were closely associated with increased homing of MPO^+^ polymorphonuclear neutrophils (PMNs) to inflamed ankles, as demonstrated by H&E staining and MPO immunohistochemistry (**Figure 1A**). K/BxN serum-induced RA induced mild synovial thickening and focal MPO^+^ inflammatory infiltration on Day 1 after the onset of arthritic joint inflammation. By Day 3, prominent acute synovitis characterized by diffuse MPO^+^ PMN infiltration, edema, and superficial bone erosions was observed in the inflamed ankles. On Day 8, the number of MPO+ PMNs and amount of edema decreased, and the amount of granulation tissue increased, leading to pannus formation. In sharp contrast, control serum-injected healthy animals showed intact joints, bone, and synovial membranes without inflammation or damage to cartilage or bone (**Figure 1A**). Next, we isolated the inflammatory cell infiltrates, presumably MPO^+^ PMNs, from the ankle lavage of RA mice at 1 d, 3 d, 6 d, and 8 d after induction of K/BxN serum-induced RA and from control serum-injected healthy control mice and characterized them by flow cytometry. We identified two subsets of MDSCs: CD11b^+^Ly6C^med^Ly6G^+^ PMN-MDSCs and CD11b^+^Ly6C^high^Ly6G^-^ M-MDSCs (**Figure 1B**). During RA progression, the numbers of CD11b^+^ cells and PMN-MDSCs increased from Day 1 to Day 6, whereas the numbers of both cellular subsets slightly decreased on Day 8 after the induction of K/BxN serum-induced RA (**Figures 1C, D**). In contrast, we did not observe significant differences in M-MDSCs between Days 1 and 8 after the induction of RA (n = 8; **Figure 1E**). Cell suspensions derived from unaffected healthy ankles of control serum-injected mice revealed almost constant percentages of CD11b^+^ (~30%) cells, PMN-MDSCs (~10%), and M-MDSCs (~2%–3%) over time (n = 8; **Figures 1C–E**). Ankle swelling in RA animals (Day 1: n = 9; Day 3–8: n = 2) constantly increased until Day 8 compared with that in controls, where the ankle thickness remained at the same level (n = 15 on Day 1, n = 4 on Days 3–8; **Figure 1F**).

**Figure 1:**
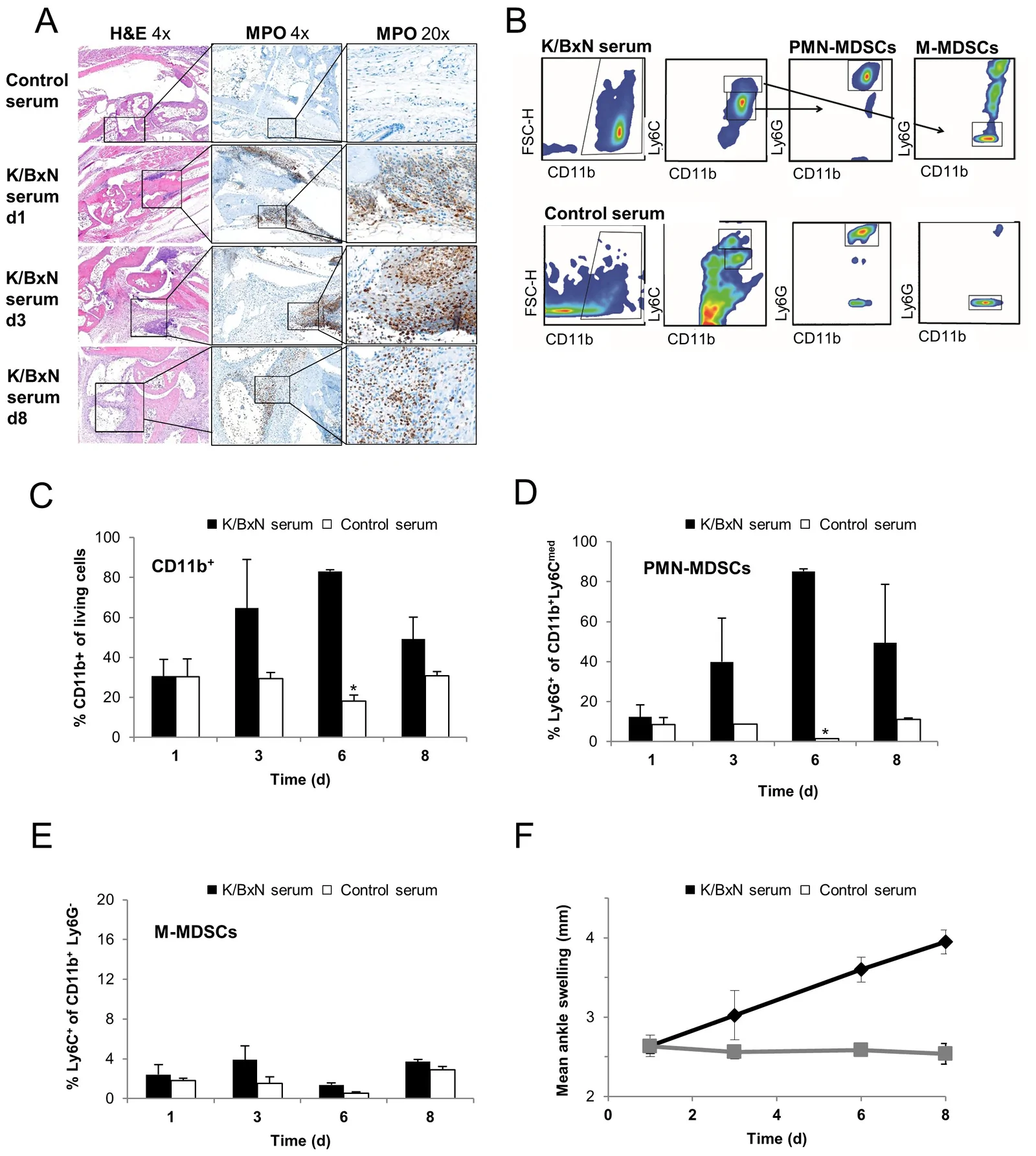
**(A)** H&E staining and MPO immunohistochemistry from arthritic ankles derived on Days 1, 3, and 8 after induction of K/BxN serum-induced RA. **(B)** Gating strategy used in the flow cytometry analysis of PMN-MDSCs and M-MDSCs. **(C–E)** Flow cytometry analysis of CD11b+ cells, PMN-MDSCs, and M-MDSCs in BALB/C mice on Days 1, 3, 6, and 8 after induction of K/BxN serum-induced RA (Day 1 and Day 3: n = 3 (pooled hind legs), Day 6: n = 2 (pooled hind legs), and Day 8: CD11b^+^ n = 6). **(F)** Ankle swelling of RA and control mice after induction of K/BxN serum-induced RA (both hind legs; Day 1 K/BXN: n = 9 control: n = 15; Day 3 K/BXN: n = 2 control: n = 4; Day 6 K/BXN: n = 2 control: n = 4; Day 8 K/BXN: n = 2 control: n = 4).

To prove the functionality of the PMN-MDSCs isolated from RA ankles, we performed a CFSE suppression assay. The *in vitro* proliferation rate of CD3/CD28 mAb-stimulated T cells isolated from the spleens of naive BALB/c mice was 37.6%, whereas incubation at a 1:1 ratio of T cells to RA-PMN-MDSCs clearly inhibited T-cell proliferation compared with T-cell proliferation in the absence of PMN-MDSCs (3.8% vs. 37.6%; **Figure 2A**). As expected, the suppression of T-cell proliferation was dependent on the number of PMN-MDSCs (**Figure 2B**).

**Figure 2:**
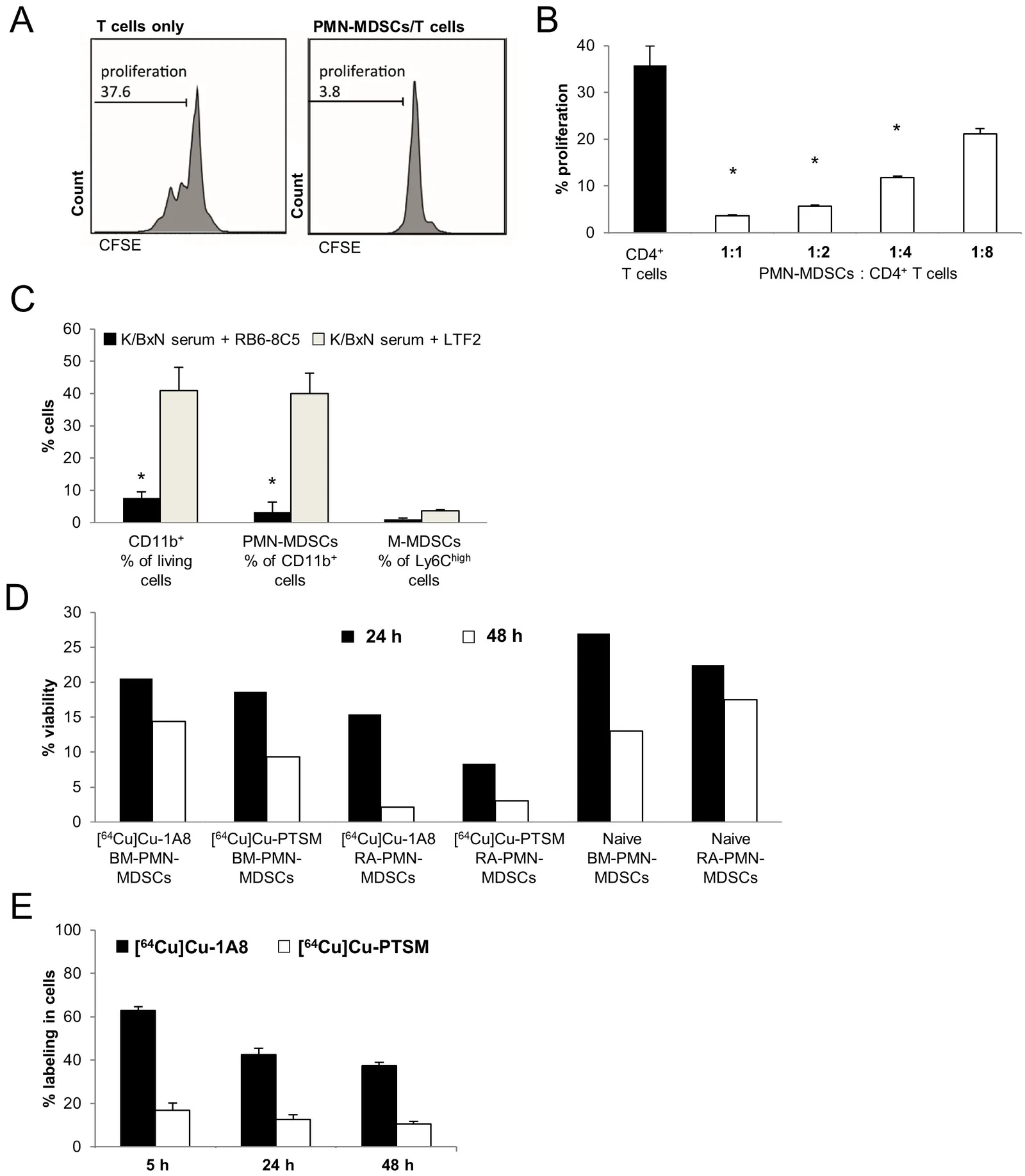
**(A, B)** T cells were labeled with CFSE and cultured for 3 days. Proliferation after the addition of PMN-MDSCs was measured by flow cytometry (T cells only, n = 4; PMN-MDSC/PMN-MDSC: CD4^+^ T cells, n = 2). **(C)** MDSCs were depleted in RA mice using the RB6-8C5 antibody or an isotype control (LTF2 antibody) on Day 6 after the induction of K/BxN serum-induced RA. Cells were subsequently isolated 24 h after ankle lavage. The number of MDSCs was measured using flow cytometry (n = 3; both hind legs were pooled). **(D)** Viability analysis of PMN-MDSCs after [^64^Cu]Cu-1A8-mAbs and [^64^Cu]Cu-PTSM labeling using flow cytometry. **(E)** Analysis of the labeling stability of PMN-MDSCs after [^64^Cu]Cu-1A8-mAbs and [^64^Cu]Cu-PTSM labeling assessed as a percentage of radiolabeled cells in the gamma counter (n = 3). * indicates significant differences.

To deplete MDSCs *in vivo*, we injected the Ly6G/Ly6C mAb RB6-8C5 on Day 5 after RA induction and sacrificed the experimental mice 24 h later. At this time point, all the mice exhibited similar amounts of ankle swelling (**Supplementary Figure 1**). Depletion of myeloid cells via the Ly6G/Ly6C mAb RB6-8C5 significantly reduced the number of CD11b^+^ cells, PMN-MDSCs, and M-MDSCs in the ankles of RA mice compared with that in isotype mAb-injected controls (**Figure 2C**).

### Viability and labeling stability of PMN-MDSCs

Next, we labeled PMN-MDSCs with [^64^Cu]Cu-PTSM or [^64^Cu]Cu-1A8 mAbs on the basis of protocols established in previous studies (19, 23). At 24 h after radiolabeling with [^64^Cu]Cu-PTSM and [^64^Cu]Cu-1A8 mAbs, ~20% of the BM-PMN-MDSCs were viable. The viability of the RA-PMN-MDSCs was slightly lower after labeling with ^64^Cu-1A8 mAbs but decreased drastically after labeling with [^64^Cu]Cu-PTSM. A further decrease in PMN-MDSC viability was observed at 48 h after radiolabeling with both [^64^Cu]Cu-PTSM and [^64^Cu]Cu-1A8 mAbs (**Figure 2D**).

Furthermore, we tested the labeling stability 5–48 h after the radiolabeling procedure. At 5 h, we detected 63 ± 1.8% of the [^64^Cu]Cu-1A8 mAb activity compared with the activity immediately after radiolabeling, with further decrease observed at 24 h (42.6 ± 3.5%) and 48 h (37.4 ± 1.6%; **Figure 2E**). In contrast, [^64^Cu]Cu-PTSM labeling of PMN-MDSCs revealed relatively poor labeling stability at all the investigated time points (5 h–48 h; **Figure 2E**).

### *In vivo* homing patterns of PMN-MDSCs in RA

[^64^Cu]Cu-PTSM- or [^64^Cu]Cu-1A8 mAb-labeled cells derived from BM and RA ankles were injected *i.v.* into RA mice 6 days after K/BxN serum injection. We subsequently evaluated the *in vivo* homing patterns by PET/MRI at 1 h and 24 h after the adoptive transfer of radiolabeled PMN-MDSCs. No significant differences in the number of PMN-MDSCs derived from RA ankles or bone marrow (RA and naive controls) were observed by flow cytometry (**Supplementary Figure 3**).

[^64^Cu]Cu-PTSM-labeled RA-PMN-MDSCs displayed a significantly increased homing to RA ankles (1 h: 4.9 ± 1.5% ID/cc; 24 h: 3.9 ± 0.4% ID/cc) compared with [64Cu]Cu-PTSM BM-PMN-MDSCs (1 h: 2.8 ± 0.4% ID/cc; 24 h: 1.9 ± 0.6% ID/cc; n = 3–4). Representative images of inflamed ankles are shown in **Figure 3A** (2× magnification).

**Figure 3:**
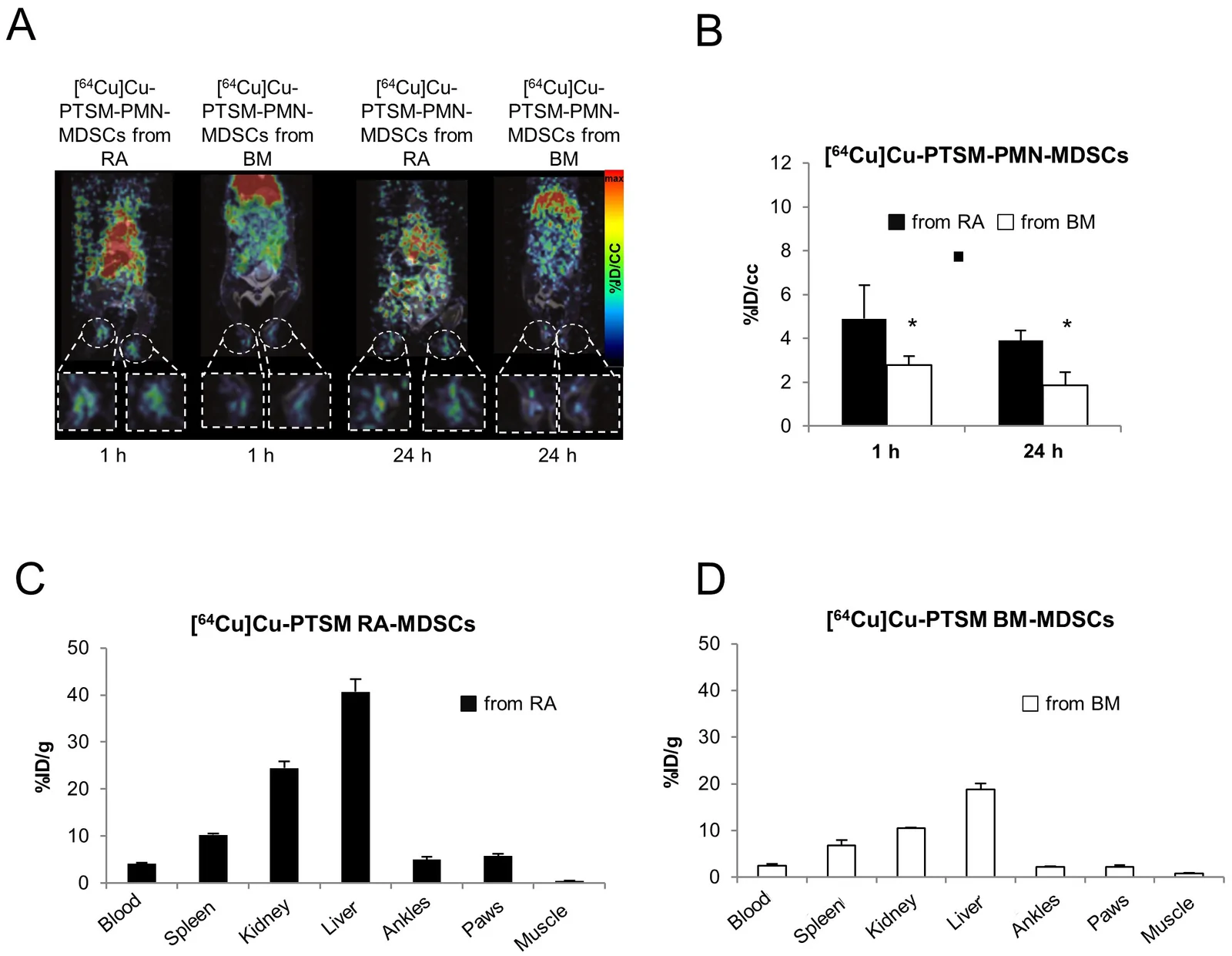
**(A)** Representative images with PET/MRI with 2× magnification of the ankles. **(B)** Quantification of the *in vivo* PET/MRI after *i.v.* injection of [^64^Cu]Cu-PTSM labeled RA- and BM-PMN-MDSCs on Day 6 after induction of RA (both hind legs; from BM n = 3; from RA n = 4); * indicates significant differences. **(C, D)** Biodistribution analysis of mice injected with [^64^Cu]Cu-PTSM-labeled RA-PMN-MDSCs and BM-PMN-MDSCs (from RA n = 2; from BM n = 3)..

*Ex vivo* biodistribution analysis at 24 h *p.i.* confirmed our *in vivo* PET/MRI data, revealing greater ankle uptake of [^64^Cu]Cu-PTSM-labeled RA-PMN-MDSCs (5.0 ± 0.6% ID/g) than [^64^Cu]Cu-PTSM-labeled BM-PMN-MDSCs (2.2 ± 0.2% ID/g; n = 2–3; **Figures 3C, D**). Nevertheless, both BM-PMN-MDSCs and RA-PMN-MDSCs exhibited relatively high liver uptake at 24 h *p.i.* (**Figures 3C, D**). Furthermore, we investigated the homing patterns of [^64^Cu]Cu-1A8 mAb-labeled BM- and RA-PMN-MDSCs in experimental mice with severe RA (**Figures 4A, B**). Similar to the results observed with [^64^Cu]Cu-PTSM-labeled PMN-MDSCs, we observed significantly enhanced homing of [^64^Cu]Cu-1A8 mAb-labeled RA-PMN-MDSCs to RA ankles compared with [^64^Cu]Cu-1A8 mAb-labeled BM-PMN-MDSCs. Representative images of inflamed ankles are shown in **Figure 4A** (2× magnification).

**Figure 4:**
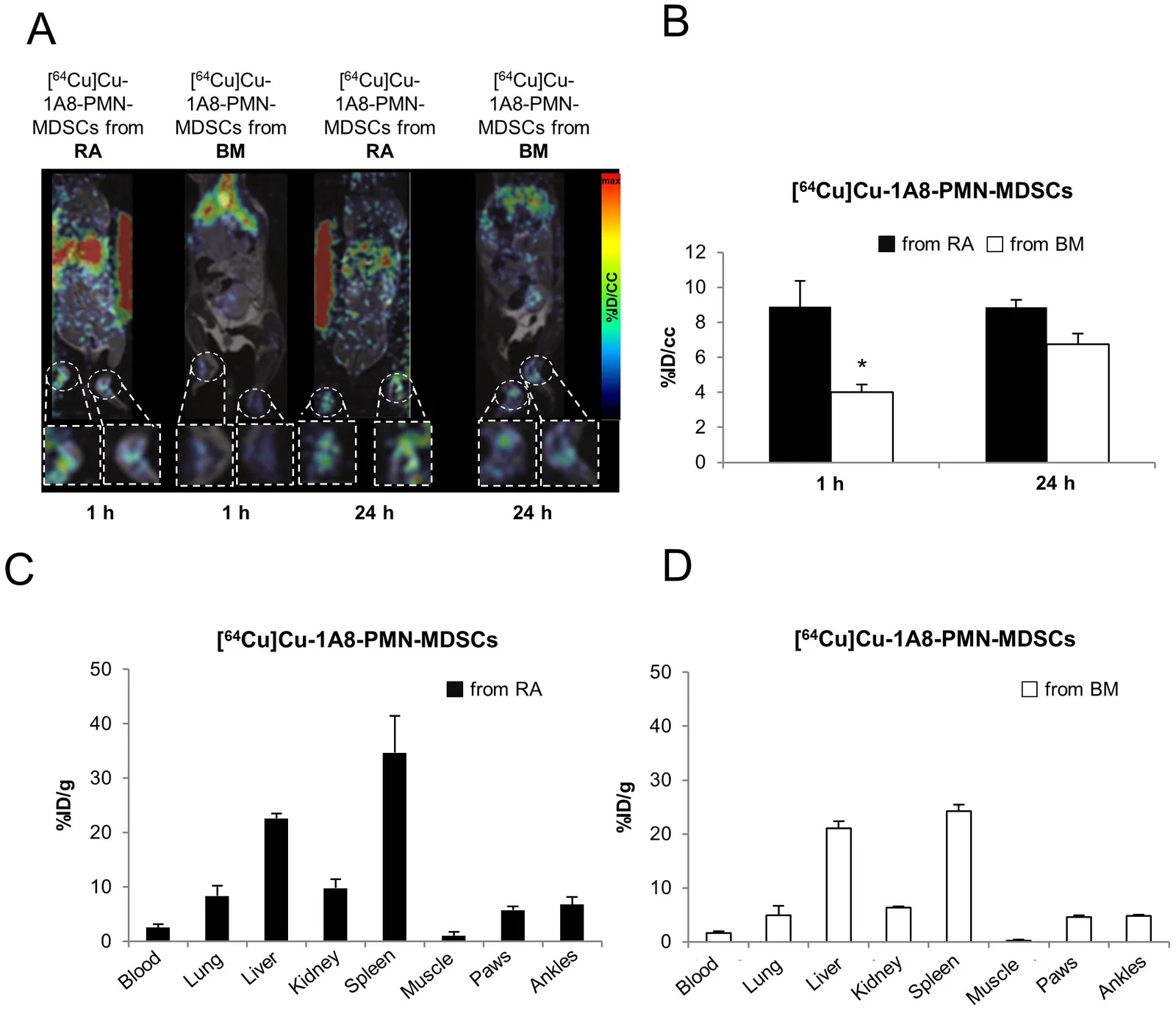
**(A)** Representative PET/MRI images with 2× magnication of the ankles. **(B)** Quantication of *in vivo* PET/MRI using [^64^Cu]Cu-1A8 mAbs labeled PMN-MDSCs from BM or RA ankles on Day 6 of RA (1 h, 24 h p.i.; n = 3); * indicates signi cant differences. **(C, D)** Biodistribution analysis of mice injected with [^64^Cu]Cu-1A8 mAbs-labeled PMN-MDSCs from RA ankles (upper panel) and BM (n = 3).

The uptake of [^64^Cu]Cu-1A8 mAb-labeled RA-PMN-MDSCs at 24 h *p.i.* was almost unchanged (8.8 ± 3.5%ID/g) compared with that at 1 h *p.i.* (8.9 ± 2.5%ID/g). In contrast, the uptake of [^64^Cu]Cu-1A8 mAb-labeled BM-PMN-MDSCs was only 4.0 ± 0.7%ID/cc after 1 h but increased to 6.7 ± 1.7%ID/cc after 24 h (**Figure 4B**).

*Ex vivo* biodistribution analysis performed 24 h after radiolabeled PMN-MDSC injection confirmed the *in vivo* PET findings. In particular, we detected a 1.4-fold increase in the homing of [^64^Cu]Cu-1A8-RA-PMN-MDSCs to the RA ankles (6.8 ± 1.4%ID/g) compared with that of [^64^Cu]Cu-1A8-BM-PMN-MDSCs (4.8 ± 0.7%ID/cc; **Figures 4C, D**).

### *Ex vivo* proteomics

To investigate the different migratory behaviors, we analyzed the quantitative proteomic profiles of BM- and RA-PMN-MDSCs by LC–MS-based proteomics (n = 5). Owing to the scarcity of cellular infiltrates, the analysis of RA-PMN-MDSCs from naive control mice was not possible. Therefore, we used only BM-MDSCs from naive mice as controls (n = 5).

By quantitative proteomic analysis of BM- and RA-PMN-MDSCs, we identified and quantified a total of 2,695 proteins. A total of 716 proteins were differentially expressed, and 186 proteins were enriched in RA-PMN-MDSCs. We deleted 530 proteins identified in BM- and RA-PMN-MDSCs, which, in comparison to those in BM-MDSCs from naive control mice, were only moderately affected (fold change <1.5, FDR <0.05). The detailed quantitative data are provided in the **Supplementary Material**. Principal component analysis (PCA) revealed clear segregation between the proteome signatures of the different groups (**Figure 5A**). Pathway analysis (30) of the proteome signature using the Functional Reactome database revealed enrichment of pathways involved in extracellular matrix organization, integrin cell surface interactions, and ECM-proteoglycans in the RA-PMN-MDSCs (**Figure 5B**). Neutrophil degranulation pathways were significantly upregulated in both RA-PMN-MDSCs and BM-PMN-MDSCs (**Figure 5B**).

**Figure 5:**
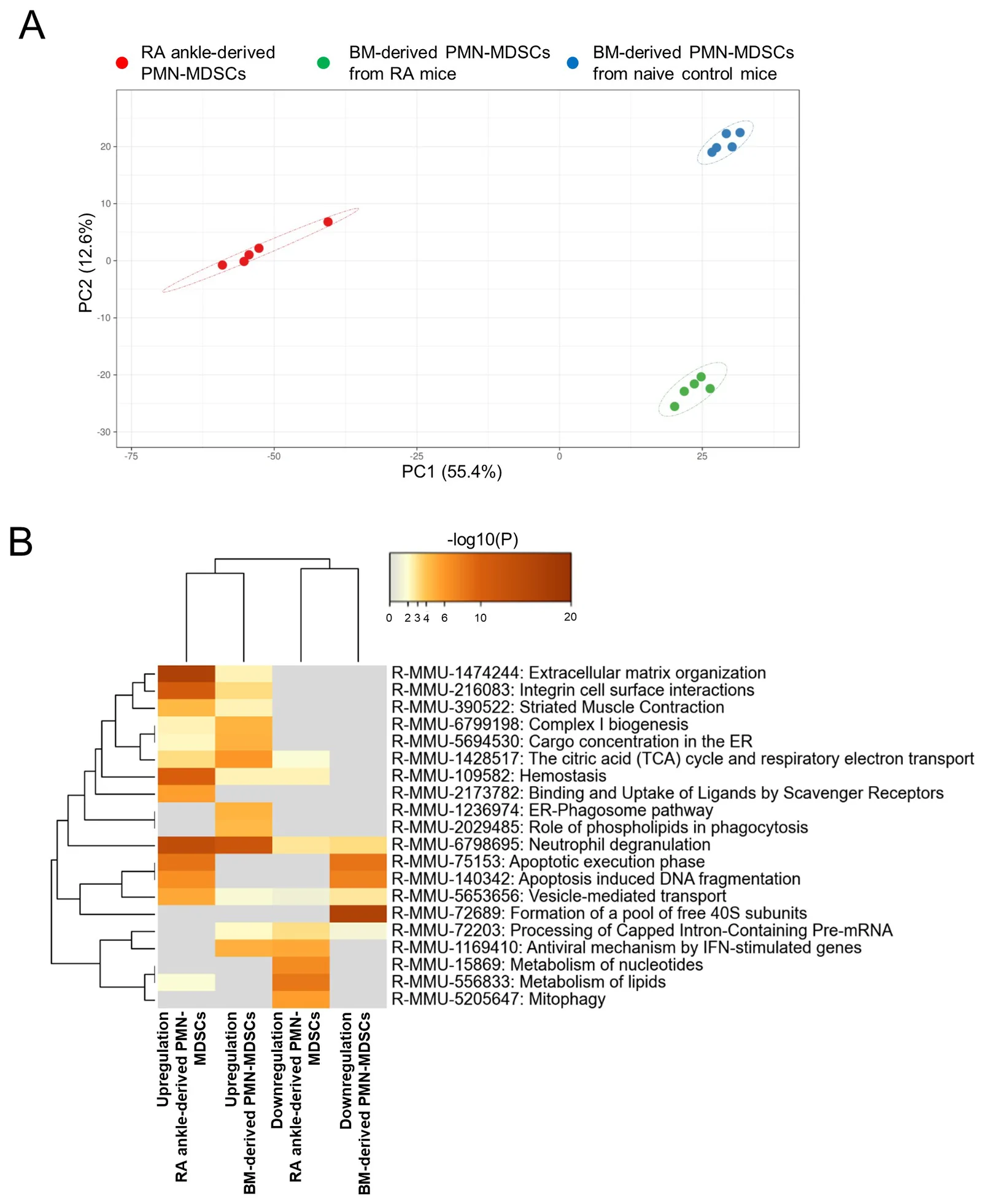
**(A)** Principal component analysis of log2 label-free quantification intensities (LFQ) of protein abundance in RA-PMN-MDSCs, BM-PMN-MDSCs, from RA mice and BM-PMN-MDSCs from naive controls. A 95% probability confidence ellipsoid was plotted around the biological replicates from each group. **(B)** Functional pathway analysis against the Reactome database revealed upregulated pathways involved in extracellular matrix organization, integrin cell surface interactions, and ECM-proteoglycans in the proteome of RA-PMN-MDSCs but not in that of BM-PMN-MDSCs. Pathways involved in neutrophil degranulation were significantly upregulated in both RA-PMN-MDSCs and BM-PMN-MDSCs.

### *In vivo* detection of Ly6G^+^ myeloid cells in RA

To further evaluate the distribution patterns of Ly6G^+^ myeloid cells *in vivo*, we *i.v.* injected 10 ± 0.1 MBq of [^64^Cu]Cu-1A8 mAbs into animals with RA on Day 6 after induction of RA with K/BxN serum and monitored [^64^Cu]Cu-1A8 mAb uptake by PET/MRI at 1 h–48 h *p.i*. To confirm the Ly6G specificity of [^64^Cu]Cu-1A8 mAb PET, we injected RA mice with 100 µg of non-radiolabeled 1A8 mAbs *i.p.* 24 h before [^64^Cu]Cu-1A8 mAb injection and determined [^64^Cu]Cu-1A8 mAb uptake in RA ankles at 1 h–48 h *p.i.* (**Figures 6A, B**). A dose of 100 µg of non-radiolabeled antagonistic 1A8 mAbs can block the accumulation of or even decrease the abundance of Ly6G^+^ myeloid cells *in vivo* (31–35).

**Figure 6:**
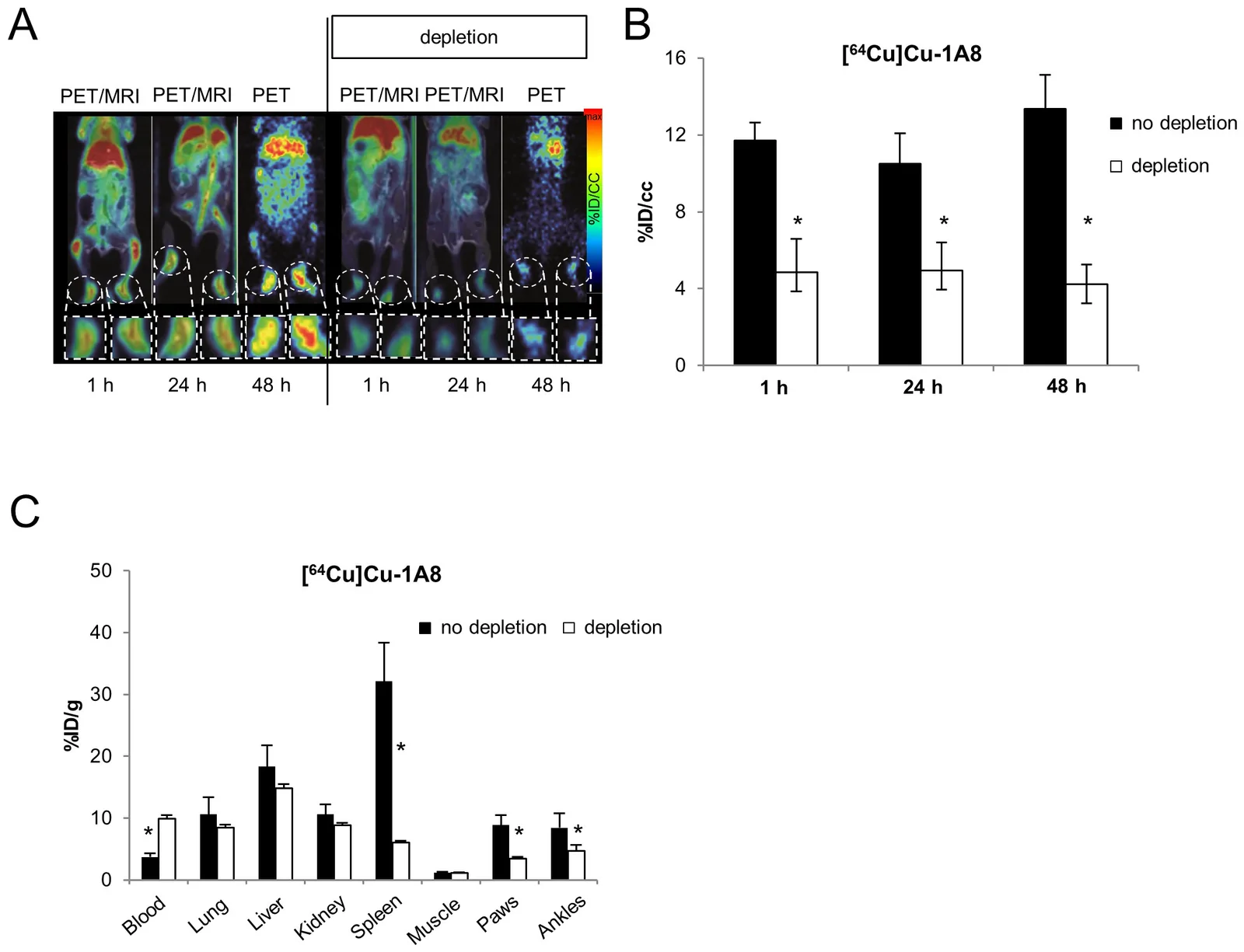
**(A)** Representative images with PET/MRI with 2× magnification of the ankles. **(B)** Quantification of *in vivo* [^64^Cu]Cu-1A8 mAbs PET results at 1 h, 24 h, and 48 h p.i. on Day 6 of RA. To investigate the specificity, depletion using 100 µg of 1A8 was performed 24 h before tracer injection; * indicates a significant difference. **(C)** Ex vivo biodistribution analysis of [^64^Cu]Cu-1A8 mAbs at 48 h p.i. with and without depletion (n = 3)..

We detected increased [^64^Cu]Cu-1A8 mAb uptake in the RA ankles at 1 h–48 h *p.i.* (1 h: 11.7 ± 1.0%ID/cc; 24 h: 10.5 ± 1.6%ID/cc; 48 h: 13.3 ± 1.8%10/cc; n = 3; **Figure 6B**), whereas depletion with non-radiolabeled Ly6G mAbs successfully inhibited [^64^Cu]Cu-1A8 mAb uptake in the RA ankles at 1 h–48 h (1 h: 4.8 ± 1.7%ID/cc; 24 h: 4.9 ± 1.5%ID/cc; 48 h: 4.2 ± 1.0%10/cc; n = 3; **Figure 6B**). *Ex vivo* biodistribution analysis confirmed the *in vivo* PET results and revealed drastically reduced [^64^Cu]Cu-1A8 mAb uptake after depletion not only in the RA ankles but also in the hind paws and the spleen (**Figure 6C**). As expected, no effect was observed within the muscle, whereas [^64^Cu]Cu-1A8 mAb activity within the blood increased (**Figure 6C**).

## Discussion

MDSCs are a heterogeneous group of myeloid cells whose common feature is their suppressive role in immune responses. Here, we developed and evaluated strategies for the non-invasive monitoring of PMN-MDSC migration by *in vivo* PET/MRI during experimental RA, which offers the potential to explore new mechanistic perspectives on autoimmune inflammation. The specific homing patterns of PMN-MDSCs in arthritic joints are dependent on disease-associated functional adaptations, which were identified by proteomic analyses. Typically, MDSCs are differentiated into M-MDSCs and PMN-MDSCs (also called G-MDSCs (3)), but the differences in their functionality have not been fully elucidated (1, 8). Here, we demonstrated that T-cell-suppressive PMN-MDSCs are the predominant cell population in arthritic ankles in K/BxN serum-induced RA (**Figures 1**, **2**). These findings are in line with the results of studies using experimental proteoglycan‐induced arthritis (36) and in patients with RA (37), in which infiltrating PMN-MDSCs were identified in the synovial fluid.

To investigate the functional role of inflammatory cells in the dynamic course of diseases, we evaluated the feasibility of *in vivo* PET/MRI to investigate the homing patterns of PMN-MDSCs in RA. *In vitro* [^64^Cu]Cu-PTSM labeling was previously established by our group for tumor antigen-specific Th1 cells in cancer immunotherapy (23). Lipophilic [^64^Cu]Cu-PTSM crosses the cell membrane by passive diffusion, followed by binding of the dissociated ^64^Cu(I) to intracellular proteins (38). Both approaches, [64Cu]Cu-1A8 mAb labeling and [64Cu]Cu-PTSM labeling, induced a drastic decrease in the number of viable RA-PMN-MDSCs observed 48 h after labeling, most likely due to radiotoxicity induced by the Auger electrons of [^64^Cu]Cu (**Figure 2D**). The labeling stability using [^64^Cu]Cu-1A8 mAbs was clearly favorable compared with that of [^64^Cu]Cu-PTSM, which is less stable than radiolabeling with an mAb (such as [^64^Cu]Cu-1A8 mAbs) (19, 23). The ~2-fold higher liver uptake with [^64^Cu]Cu-PTSM likely reflects hepatic clearance of free tracer (**Figures 3C, D** vs. **Figures 4C, D**), whereas greater splenic accumulation of [^64^Cu]Cu-1A8 mAb-PMN-MDSCs may result from improved labeling stability or antibody binding to other Ly6G-expressing splenic cells (**Figure 2E**).

In addition, non-specific accumulation of dead PMN-MDSCs or cell debris at inflammatory sites is unlikely, as immune-cell migration requires active chemotaxis, selectin-mediated rolling, and ICAM-1/VCAM-1-dependent adhesion (39). Thus, we can largely exclude the migration of dead or dying cells into inflamed ankles. The differences in tracer uptake between [^64^Cu]Cu-PTSM-PMN-MDSCs and [^64^Cu]Cu-1A8-PMN-MDSCs are most likely explained by differences in viability (**Figures 3B**, **4B**).

The increased homing of PMN-MDSCs isolated from RA ankles to RA ankles compared with that of PMN-MDSCs from the BM may indicate that the polarization of MDSCs induced by RA enables increased infiltration into inflammatory tissue. Nevertheless, no specific data on this phenomenon regarding PMN-MDSCs in K/BxN serum-induced RA are available in the literature despite extensive published results on the migratory behavior of other myeloid cells, such as neutrophils and macrophages (40, 41). If the differences in viability between RA-derived and BM-derived MDSCs influenced the uptake of the radiolabeled cells, a lower uptake of RA-derived MDSCs in the RA ankles would be expected because of dead cells. Instead, the results in **Figures 2** and **3** indicate that the uptake of RA-derived MDSCs in the RA ankles was greater than that of BM-derived MDSCs.

Our proteome analysis revealed distinct differences in protein expression between RA-PMN-MDSCs and BM-PMN-MDSCs, indicating functional differences among MDSCs from different tissue origins, which were isolated using the same phenotypic markers. In particular, integrins are critically involved in cellular migration and adhesion in RA and other diseases (42). Sugahara et al. showed that α9-integrin blockade suppressed the development of experimental collagen-induced arthritis (43).

Neutrophil subsets, including PMN-MDSCs, are subjected to constantly changing functional diversity, which depends on the inflammatory environment and the tissue from which these cells are isolated (44). Grieshaber-Bouyer et al. reported distinct transcriptional signatures in neutrophils derived from BM or RA ankles during GPI-induced arthritis (45), which were also apparent in our proteome analysis (**Figure 5**). These gene signatures, consisting of multiple differentially expressed genes, drive the migratory behavior of BM- and RA ankle-derived PMN-MDSCs. These results provide new hypotheses for subsequent studies to identify mechanisms of cell migration in PMN-MDSCs derived from different tissues, which might be useful as therapeutic targets in the future.

To differentiate specific from non-specific uptake of the [^64^Cu]Cu-1A8 mAb, we depleted neutrophils, including PMN-MDSCs using unlabeled 1A8 mAb (**Figure 6**). Wang et al. reported blood neutrophil depletion at 24 h, but not 4 h, after 5 µg 1A8 mAb, with no bone marrow depletion (31). Thus, significant PMN-MDSC depletion is unlikely within 1 h of [^64^Cu]Cu-1A8 mAb administration (31). Its effect on neutrophils already accumulated in inflamed tissues remains unclear; in our model, these cells were already abundant in severely inflamed ankles. Nevertheless, at high doses, such as those used for depletion experiments (100 μg), the Ly6G antibody 1A8 depletes neutrophil cell populations, including PMN-MDSCs (46). Depletion by 1A8 at higher doses is mediated by macrophage-dependent cellular phagocytosis, not complement-dependent cytotoxicity (47, 48). Although [^64^Cu]Cu-1A8 targets Ly6G-expressing granulocytes beyond PMN-MDSCs, including neutrophils, its uptake pattern closely matched that of adoptively transferred PMN-MDSCs labeled *in vitro* with [^64^Cu]Cu-1A8 mAb (**Figure 4**).

Boivin et al. used an identical clone (1A8) to demonstrate the internalization of the 1A8 mAb (49). Thus, assuming that our [^64^Cu]Cu-1A8 mAbs are incorporated into Ly6G-expressing cells, we are convinced that our technique is also applicable for tracking Ly6G-expressing cells. In addition to the lower experimental effort, the advantage of [^64^Cu]Cu-1A8 mAbs as radiotracers is the potential for clinical translation, where safety restrictions and regulatory hurdles are immense for cell transfers in diagnostic procedures.

In conclusion, PET/MRI-based imaging enables non-invasive monitoring of PMN-MDSC migration during experimental RA and provides new insights into the role of these cells in autoimmune inflammation. These findings establish PET-based PMN-MDSC imaging as a promising tool for investigating myeloid cell dynamics and developing PMN-MDSCs as biomarkers and therapeutic targets for autoimmune diseases.

## Data Availability

The original contributions presented in this study are publicly available. The data supporting the findings of this study are provided within the article and its Supplementary Information. The proteomics datasets, including LC–MS raw data files, database search engine outputs, associated metadata, and search parameters required to reproduce the results, have been deposited in the MassIVE repository (https://massive.ucsd.edu/; accession number: MSV000103531) and are also accessible through ProteomeXchange (https://proteomecentral.proteomexchange.org/; accession number: PXD085378). Additional raw data supporting the conclusions of this article will be made available by the authors upon request, without undue reservation.
