# Supplementary material for: In vivo monitoring of polymorphonuclear myeloid-derived suppressor cells in experimental rheumatoid arthritis
Source: Front Immunol. 2026 Oct 15;17:1909885. doi: 10.3389/fimmu.2026.1909885 (PMC13716723; doi:10.3389/fimmu.2026.1909885)
Supplement: Supplementary file 1 [file DataSheet1.docx]

**Supplementary Figure 1**

Ankle swelling after application of the anti-RB6-8C5 antibody or isotype control (LTF2 antibody) on Day 5 after the induction of RA with K/BxN serum.

**Supplementary Figure 2**

**Timeline of RA induction and PET investigations**. RA mice injected with [^64^Cu]Cu-PTSM-labeled RA- and BM-PMN-MDSCs or [^64^Cu]Cu-1A8-mAb-labeled RA- and BM-PMN-MDSCs underwent PET/MRI at 1 h and 24 h *p.i.* RA mice injected with [^64^Cu]Cu-1A8-mAbs underwent PET/MRI at 1 h, 24 h and 48 h *p.i.*

**Supplementary Figure 3**

**Gating strategy used for flow cytometry analysis** of cells isolated from **A.** the bone marrow of control mice (n=6), **B**. the bone marrow of K/BxN serum-induced RA mice (n=4), or **C**. the ankle joints of K/BxN serum-induced RA mice (n=4). **A+B**. Bone marrow cells were isolated from the femur and tibia and differentiated for 6 days in the presence of 40 ng/mL murine GM-CSF and 40 ng/mL murine IL-6. Ly6G^+^ cells were subsequently isolated by magnetic-activated cell sorting (MACS). **C**. Cells were flushed from the RA ankles, and Ly6G^+^ cells were isolated by MACS. **D.** Flow cytometric identification of Ly6G^+^ Ly6C^med^ PMN-MDSCs within the parental CD45^+^ CD11b^+^ population. The data are presented as the mean ± SD.

**Supplementary Table 1**

| Antigen | Fluorochrome | Clone | Manufacturer |
| --- | --- | --- | --- |
| CXCR4 | V450 | 2B11 | eBioscience |
| CD45.2 | BD V500 | 104 | BD Biosciences |
| CD244.2 | FITC | eBio244F4 | eBioscience |
| Ly6G | PE | 1A8 | BD Biosciences |
| Ly6C | APC | AL-21 | BD Biosciences |
| CD115 | PE-Cy7 | AFS98 | eBioscience |
| CD11b | APC-eFluor780 | M170 | eBioscience |

Antibody panel used for flow cytometry analysis.
